# Triazine-Acceptor-Based Green Thermally Activated Delayed Fluorescence Materials for Organic Light-Emitting Diodes

**DOI:** 10.3390/ma12162646

**Published:** 2019-08-20

**Authors:** Ramanaskanda Braveenth, Kyu Yun Chai

**Affiliations:** Division of Bio-Nanochemistry, College of Natural Sciences, Wonkwang University, Iksan City 570-749, Chonbuk, Korea

**Keywords:** Triazine, TADF, Delayed time, green OLED

## Abstract

High-efficiency thermally activated delayed fluorescence (TADF) is leading the third-generation technology of organic light-emitting diodes (OLEDs). TADF emitters are designed and synthesized using inexpensive organic donor and acceptor derivatives. TADF emitters are a potential candidate for next-generation display technology when compared with metal-complex-based phosphorescent dopants. Many studies are being conducted to enhance the external quantum efficiencies (EQEs) and photoluminescent quantum yield of green TADF devices. Blue TADF reached an EQE of over 35% with the support of suitable donor and acceptor moieties based on a suitable molecular design. The efficiencies of green TADF emitters can be improved when an appropriate molecular design is applied with an efficient device structure. The triazine acceptor has been identified as a worthy building block for green TADF emitters. Hence, we present here a review of triazine with various donor molecules and their device performances. This will help to design more suitable and efficient green TADF emitters for OLEDs.

## 1. Introduction

Organic light-emitting diodes (OLEDs) and the application of organic materials in emitting technology have attracted much attention from industrial and research communities since 1987. The advantages of OLED technology, such as its light weight, image quality, high contrast, fast response time, thin film, and wide-view angle, have made it a potential candidate in commercial applications instead of liquid crystal displays (LCDs) [1,2,3,4,5]. Moreover, OLED displays can be fabricated on foldable and bendable substrates, thus making them a leading type of next-generation display. OLEDs have received considerable attention as an energy-efficient technology because they do not require any backlighting support [6,7,8]. OLED technology has developed from single- to multilayer devices across three generations of dopant materials. Multilayer OLED devices consist of several layers between an anode and a cathode, including a hole-injection layer (HIL), a hole-transporting layer (HTL), an electron-blocking layer (EBL), an emission layer (EML), a hole-blocking layer (HBL), an electron-transporting layer (ETL), and an electron-injection layer (EIL). The emission layer is made of two components, namely, host and dopant materials. The dopant material, at a suitable doping concentration, is usually doped with a high–triplet energy host material to support effective energy balance and emit colors by a proper charge recombination [9,10,11,12,13,14,15,16,17,18,19]. 

The spin rule explains the singlet and triplet emission possibilities of OLED dopants, where 25% are only responsible for singlet emission while 75% are from the triplet state. First-generation fluorescent emitters harvest only singlet emission with an internal quantum efficiency (IQE) of 25%. The remaining 75% of the IQE does not hold any emission responsibility for colors [20,21,22,23,24,25]. Phosphorescent OLEDs (PhOLEDs) were developed by using iridium- and platinum-based noble metals to enable the 100% IQE by activating the intersystem crossing (ISC) pathway [26,27,28,29,30,31,32]. The efficiency of PhOLEDs was enhanced further by developing various host materials. Host materials are responsible for supplying energy to the dopant while preventing energy flow back from the dopant [33,34,35,36,37]. The major disadvantage of PhOLEDs is that they require the use of expensive noble metals to form a metal–ligand complex, which produces toxic waste [38,39,40,41,42,43]. 

Current studies are exploring new technologies using metal-free dopants and a type of emission referred to as thermally activated delayed fluorescence (TADF). This TADF mechanism can also achieve 100% IQE by activating an efficient up-conversion process of reverse intersystem crossing (RISC) from an excited triplet state to an excited singlet state [44,45,46,47,48,49,50,51]. The TADF concept is based on organic molecules with suitable donor–acceptor building blocks. Donor and acceptor moieties present in the same molecule enable intramolecular charge transfer (ICT) with a small energy difference between singlet and triplet excited states to allow an effective RISC process to harvest singlet emissions through converting triplets to singlets. Moreover, clear separation between highest occupied molecular orbitals and lowest unoccupied molecular orbital (HOMO and LUMO) distribution, twisted molecules, and a phenyl linker between donor and acceptor support an effective TADF mechanism that can achieve highly efficient device performances [52,53,54,55,56,57,58,59,60]. 

Many donor–acceptor-based structures have been reported for TADF emitters. The most commonly reported donor moieties are carbazole, diphenylamine, acridine, phenoxazine, phenothiazine, and their derivatives. The electron-withdrawing groups of cyano, boron, pyridine, pyrimidine, triazine, sulfone, dicyanofluorene, pyrazine, and ketone have been employed as acceptor moieties [61,62,63,64,65,66]. Suitable donor and acceptor combinations create efficient TADF emitters and help to decrease the singlet–triplet energy gap, which increases the rate constant of RISC. According to Boltzmann’s equation, increasing the rate of ISC and RISC helps to decrease the delayed fluorescence time. Over the past few years, great advances have been made in the efficiencies of red, green, and blue color development [67,68].

The external quantum efficiencies (EQEs) of red, green, and blue TADF OLEDs have made great progress along with new molecular designs. Red emitters suffer due to low band-gap energy and longer wavelengths near the IR region. Recently, green TADF emitters have received much attention because of their molecular design and efficiency enhancement. Strong-donor–weak-acceptor, weak-donor–strong-acceptor, and moderate-donor–moderate-acceptor combinations were identified and applied to green TADF molecular constructions [66,69,70,71]. Acridine, phenoxazine, and phenothiazine are strong donor groups, and boron and sulfone derivatives are strong withdrawing groups. The color purity of emitters was controlled according to the type and number of donor moieties attached with different positions of linker units present between donor and acceptor moieties. Among the reported cyano-acceptor-based green TADF emitters, 4CzIPN exhibited good device characteristics. The EQEs of 4CzIPN green emitters were 14%, 19.3%, 21.8%, 26.5%, 27.5%, 26.7%, 28.6%, 29.6%, and 31.2% with various host materials of mCBP, CBP, 4CN34BCz, mCPSOB, mCP:TSPO1, DCzDCN, mCP:BmPyPb, mCP:B3PYMPM, and 3CzPFP, respectively [72,73,74,75,76,77,78,79]. The weak electron acceptor of the pyrazine moiety with different donor-derivative-based green TADF emitters exhibited low device characteristics, but pyrimidine-based Ac-HPM, Ac-PPM, and Ac-MPM revealed considerable EQEs of 20.9%, 19.0%, and 24.5%, respectively [80]. A thioxanthone acceptor unit with carbazole (TXO-PhCz) and triphenylamine (TXO-TPA) donor derivatives showed EQEs of 21.5% and 18.5%, respectively, and the efficiencies were better than those of pyrazine-based green TADF emitters [59]. The stable and moderate electron-withdrawing triazine molecule showed great efficiencies and improved device stabilities. The boron-acceptor-based green TADF emitters PXZ-Mes_3_B and 2DAC-Mes_3_B revealed EQEs of 22.8% and 21.6%, respectively, which were higher than those of thioxanthone-based green emitters [69]. A triazine acceptor with different donor moieties of TmCzTrz, DMAC-TRZ, TRZ-DDPAc, and DACT-II had excellent EQEs of 25.5%, 26.5%, 27.3%, and 29.6%, respectively, and the current efficiencies of acridine-donor-based DMAC-TRZ and TRZ-DDPAc were 66.8 and 62.8 cd/A, respectively [81,82,83,84].

**Figure 1:**
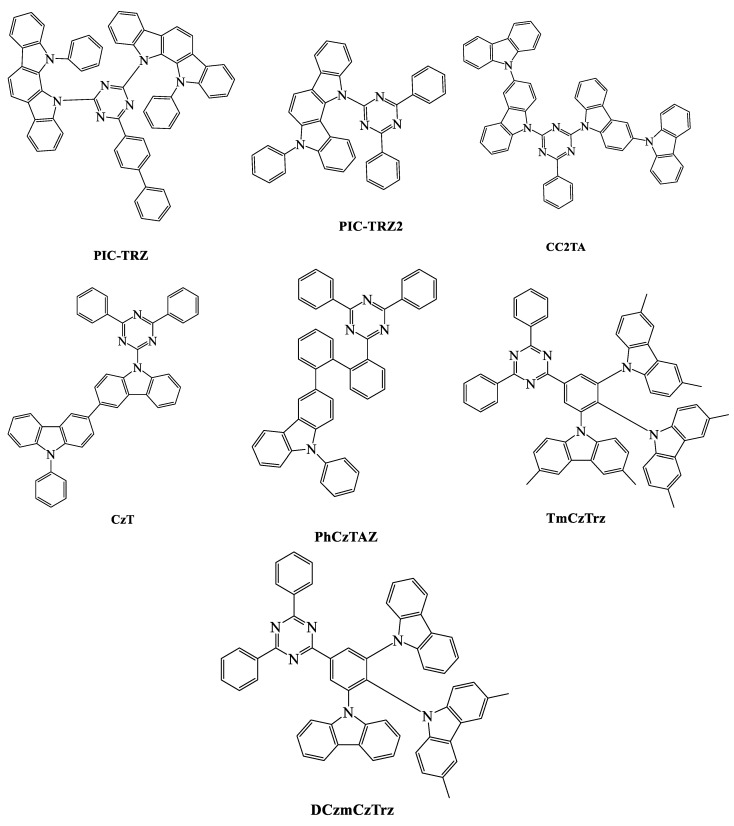
Molecular structures of triazine acceptors with carbazole donor moieties.

**Figure 2:**
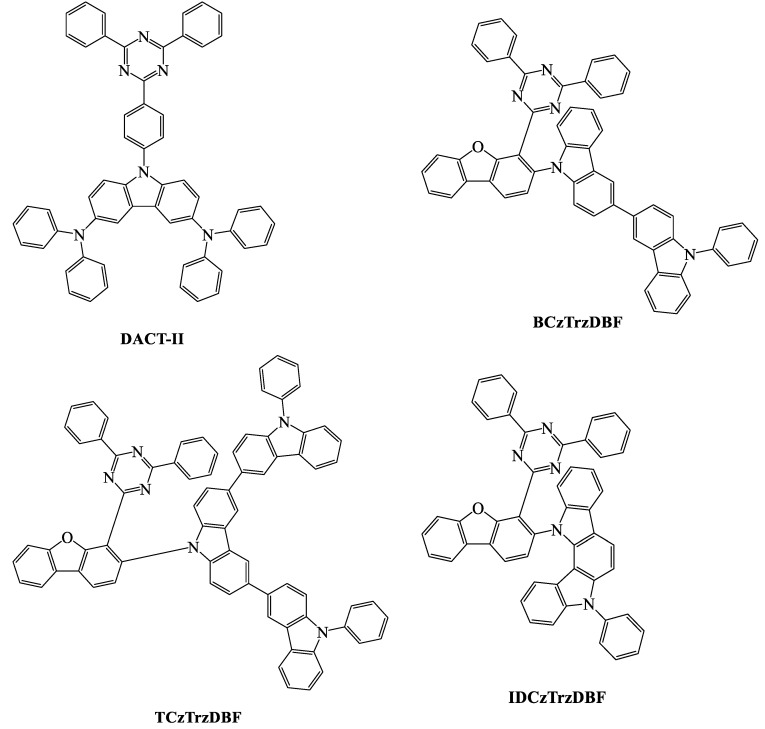

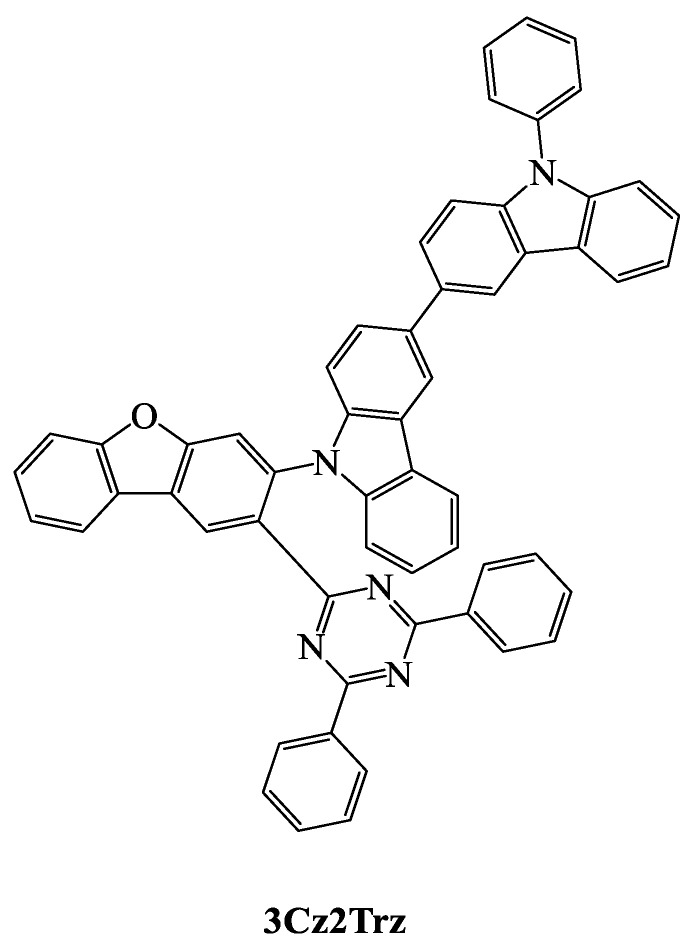
Molecular structures of triazine acceptors with carbazole, along with other donor derivatives.

**Figure 3:**
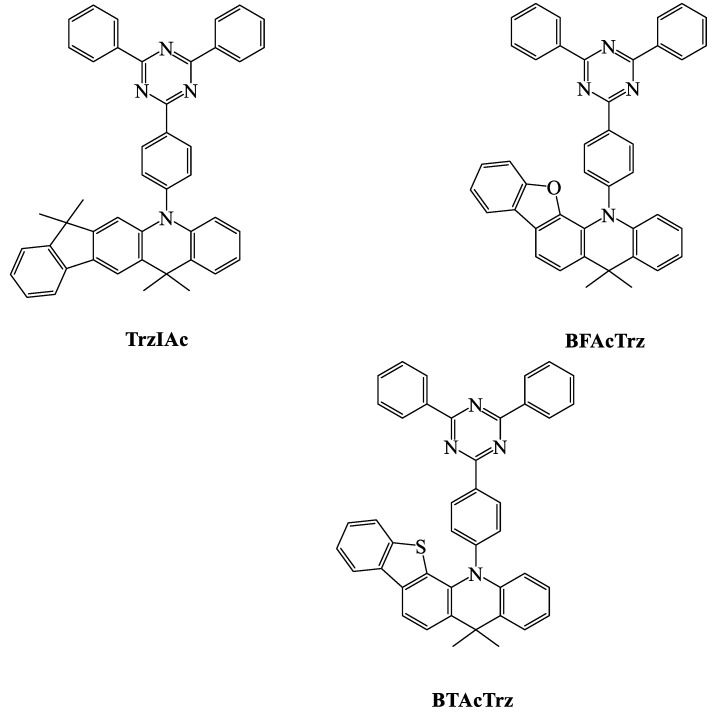
Molecular structures of triazine acceptors with acridine-based rigid donor moieties.

**Figure 4:**
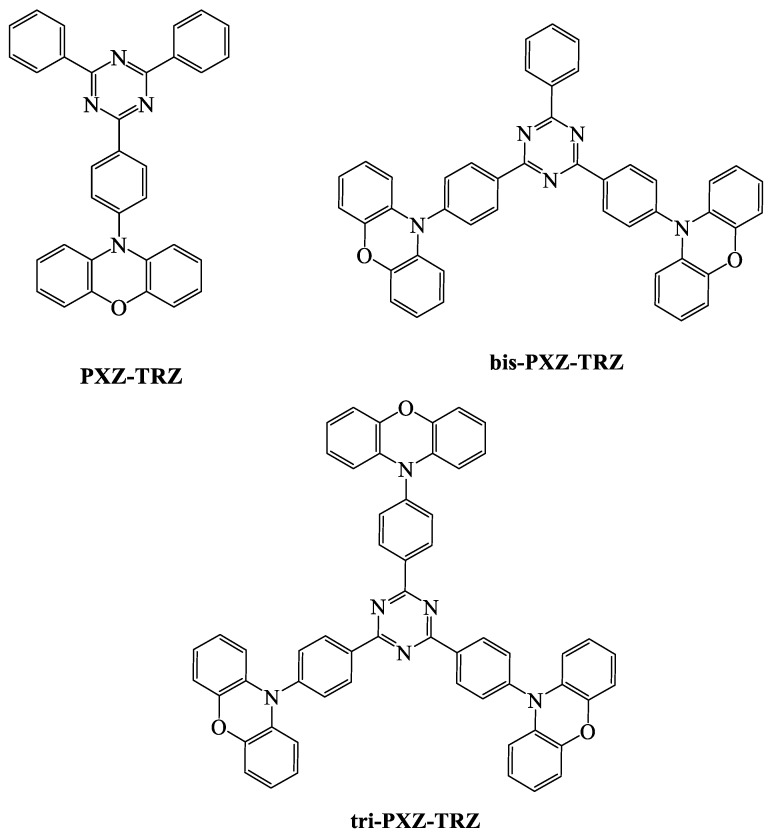
Molecular structures of triazine acceptors with phenoxazine donor moieties.

**Figure 5:**
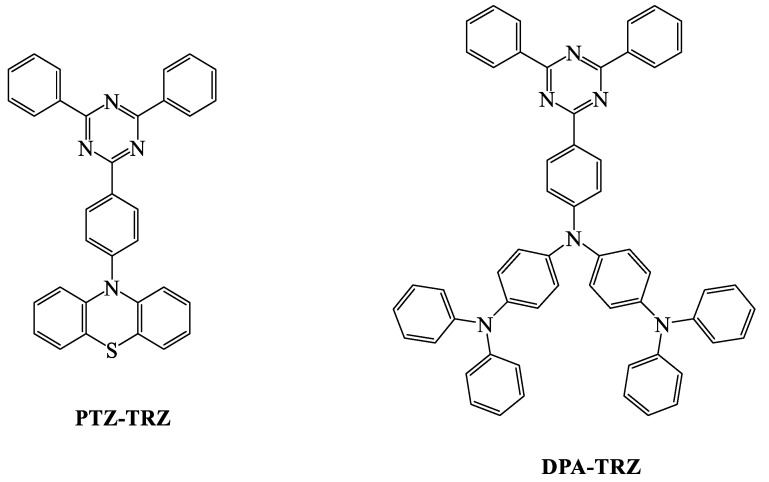
Molecular structures of triazine acceptors with phenothiazine and diphenylamine donor moieties.

**Figure 6:**
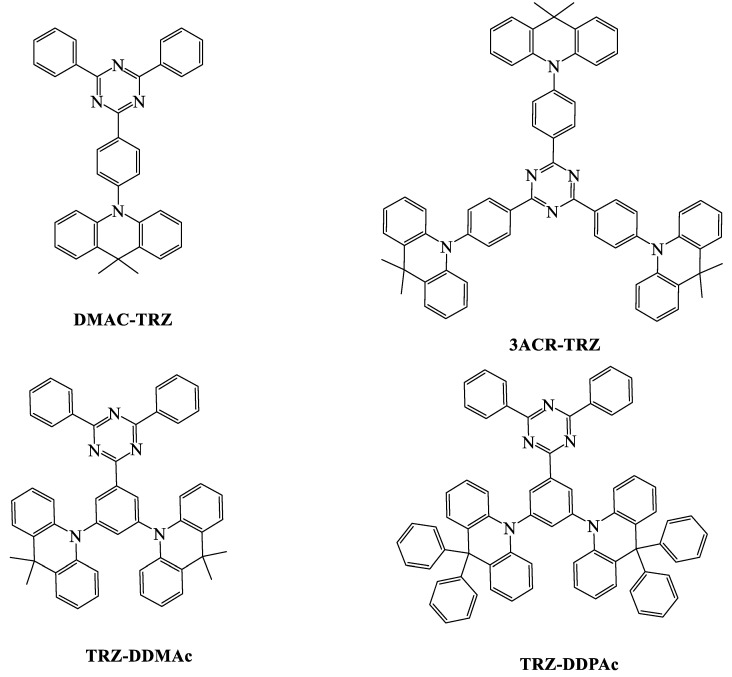
Molecular structures of triazine acceptors with acridine donor moieties.

When we compare the device efficiencies of various acceptor-based green TADF emitters, the triazine acceptor with suitable donor moieties enhances the device efficiencies and photoluminescent quantum yield (PLQY). In this review, we focus on triazine-based green TADF emitters and their device characteristics. As a moderate acceptor, triazine is an interesting derivative for green TADF emitters. Triazine-based green TADF emitters are depicted in Figure 1, Figure 2, Figure 3, Figure 4, Figure 5 and Figure 6, and their photophysical properties and device performances are summarized in Table 1 and Table 2, respectively.

## 2. Results and Discussion

The heterocyclic triazine acceptor is a well-known moiety for green TADF emitters due to its stable and moderate electron acceptability. Many studies have reported that efficient green TADF emitters were developed by replacing various donor moieties in different positions and suitable device structures, especially the host material.

12,12'-(6-([1,1'-biphenyl]-4-yl)-1,3,5-triazine-2,4-diyl)bis(11-phenyl-11,12 dihydroindolo[2,3a]carbazole) (**PIC-TRZ**) (Figure 1) was developed with two steric indolocarbazole donor units to confine the pi conjugation, which helped to reduce the singlet–triplet energy gap through clear frontier molecular orbital separation [85]. The PLQY was reported to be 39% and had a delayed fluorescence time of 230 μs. This OLED device was fabricated with mCP host material, which has a high–triplet energy of 2.91 eV, to ensure effective energy transfer from the host to the dopant. The electroluminescent emission was recorded at 500 nm and the EQE was 5.3%. The monosubstituted donor-based 12-(4,6-diphenyl-1,3,5-triazin-2-yl)-5-phenyl-5,12-dihydroindolo[3,2-a]carbazole (**PIC-TRZ2**) not only had a lower singlet–triplet energy difference of 0.02 eV compared with **PIC-TRZ** but also showed a high quantum yield of 45%. The EQE was boosted from 5.3% to 14% with an effective reverse intersystem crossing [86].

Lee et al. reported another molecule with a disubstituted bicarbazole donor derivative at the second and fourth positions of the triazine acceptor. 9,9''-(6-phenyl-1,3,5-triazine-2,4-diyl)bis((9H-3,9'-bicarbazole)) (**CC2TA**) (Figure 1) showed a low energy difference of 0.05 eV between the singlet and triplet levels, which was supported by the considerable separation between the donor and acceptor units. A photoluminescent quantum yield of 62% was recorded, while delayed fluorescence was observed at 22 μs. This OLED device was constructed using a double emission layer with host materials such as mCP and DPEPO. The double-layered host materials were responsible for opposite charge transportation, and a thin layer of DPEPO was employed to block excitons at the interface between the emission layer and the electron-transporting layer. The device exhibited an external quantum efficiency of 11% and an emission of 490 nm [87].

The carbazole-triazine based 9-(4,6-diphenyl-1,3,5-triazin-2-yl)-9'-phenyl-9H,9'H-3,3'-bicarbazole (**CzT**) (Figure 1) molecule showed a PLQY of around 40%, and the singlet–triplet energy gap was observed to be 0.07 eV. This OLED device was fabricated with the high–triplet energy host material DPEPO to ensure effective energy transfer. Also, a low concentration of 3 wt % **CzT** was applied during the fabrication process to prevent fluorescence quenching at the emission layer. The power and EQE were 9.7 lm/W and 6%, respectively, and electroluminescent emission was recorded at 520 nm. Additionally, a biphenyl link between the triazine acceptor and carbazole donor for the 3-(2'-(4,6-diphenyl-1,3,5-triazin-2-yl)-[1,1'-biphenyl]-2-yl)-9-phenyl-9H-carbazole (**PhCzTAZ**) molecule (Figure 1) displayed a higher singlet–triplet energy gap and made an impossible reverse intersystem crossing, which did not show any delayed component in photophysical evaluation [88].

Lee et al. studied triazine-based TADF emitters with an increased number of carbazole donor moieties, which were attached with a phenyl linker unit (Figure 1). 9,9',9''-(5-(4,6-diphenyl-1,3,5-triazin-2-yl)benzene-1,2,3-triyl)tris(3,6-dimethyl-9H-carbazole) (**TmCzTrz**) and 9,9'-(2-(3,6-dimethyl-9H-carbazol-9-yl)-5-(4,6-diphenyl-1,3,5-triazin-2-yl)-1,3-phenylene)bis(9H-carbazole) (**DCzmCzTrz**) revealed PLQYs of 100% and 98%, respectively, and the energy difference between the singlet and triplet states were 0.07 and 0.20 eV, respectively. The higher PLQY of the **TmCzTrz** molecule showed greater efficiencies (18.6 cd/A, 52.1 lm/W, and 25.5%) than the **DCzmCzTrz** molecule. The number of carbazole donors with even HOMO distribution increased, and the device efficiencies were enhanced [89].

The study of the 9-(4-(4,6-diphenyl-1,3,5-triazin-2-yl)phenyl)-N3,N3,N6,N6-tetraphenyl-9H-carbazole-3,6-diamine (**DACT-II**) molecule (Figure 2) showed an interesting photoluminescent quantum yield of 100%, which matched the theoretical IQE of 100%. The energy difference between the singlet and triplet states was 0.009 eV. An energy gap near zero supported a large oscillator strength and an effective RISC process. The bulky molecular structure had favorable thermal properties, and the decomposition temperature was 484 °C at 5% weight reduction. The symmetric diphenylaminocarbazole donor in this molecule played an important role in the device efficiencies. An OLED device was fabricated with a 100 nm thick 4,4′-Cyclohexylidenebis[N,N-bis(4-methylphenyl)benzenamine] (TAPC) hole-transporting layer and was doped with CBP host material. An EQE of 29.6% was recorded without any outcoupling techniques. At the same time, the device exhibited low power consumption, even at high thickness [84].

Dibenzofuran–carbazole-donor-based 9-(4-(4,6-diphenyl-1,3,5-triazin-2-yl)dibenzo[b,d]furan-3-yl)-9'-phenyl-9H,9'H-3,3'-bicarbazole (**BCzTrzDBF**), 9’-(4-(4,6-diphenyl-1,3,5-triazin-2-yl)dibenzo[b,d]furan-3-yl)-9,9’’-diphenyl-9H,9’H,9’’H-3,3’:6’,3’’-tercarbazole (**TCzTrzDBF**), and 12-(4-(4,6-diphenyl-1,3,5-triazin-2-yl)dibenzo[b,d]furan-3-yl)-5-phenyl-5,12-dihydroindolo[3,2a]carbazole (**IDCzTrzDBF**) were synthesized and used for green TADF emitters (Figure 2). A dibenzofuran unit was used as the backbone between the carbazole donor and the triazine acceptor, and a HOMO–LUMO distribution confirmed the linker unit. All three molecules exhibited PLQYs over 80%. The delayed time was recorded as 5.4, 4.4, and 2.8 μs for **BCzTrzDBF**, **TCzTrzDBF**, and **IDCzTrzDBF**, respectively. The reverse intersystem crossing rate constant of **IDCzTrzDBF** was high due to the large angle between the indolocarbazole donor and the dibenzofuran linker. **TCzTrzDBF** exhibited the highest horizontal dipole alignment ratio relative to the substrate of 0.79, which helped to bring a higher EQE of 23.5% compared with **BCzTrzDBF** (20.1%) and **IDCzTrzDBF** (12.2%). Moreover, the three-carbazole-unit-based **TCzTrzDBF** enhanced the current and power efficiencies (74.8 cd/A and 44.7 lm/W, respectively) compared with the two-carbazole-unit-based **BCzTrzDBF** (59.6 cd/A, 35.1 lm/W). Further studies were carried out by Jung et al. by changing the donor and acceptor positions with a dibenzofuran linker unit (Figure 2). 9-(4-(4,6-diphenyl-1,3,5-triazin-2-yl)dibenzo[b,d]furan-3-yl)-9'-phenyl-9H,9'H-3,3'-bicarbazole (**2Cz3Trz**) and 9-(2-(4,6-diphenyl-1,3,5-triazin-2-yl)dibenzo[b,d]furan-3-yl)-9'-phenyl-9H,9'H-3,3'-bicarbazole (**3Cz2Trz**) showed low PLQYs and device efficiencies compared with **TCzTrzDBF**. Changing the acceptor attached position and reducing the amount of carbazole did not reveal any interesting efficiency enhancements. However, a dibenzofuran linker can suppress the nonradiative mechanism when compared with the presence of a phenyl linker moiety [90,91]. 

Carbazole and its derivatives with a triazine acceptor were the subject of an interesting study on device performances. The monosubstituted indolocarbazole donor moiety **PIC-TRZ2** showed a well-separated frontier molecular orbital distribution compared with disubstituted indolocarbazole **PIC-TRZ2**, which helped to increase the EQE from 5.3% to 12.5%. However, these two molecules did not have any phenyl linker or spacer molecule between the donor and the acceptor. A bicarbazole donor and triazine acceptor without any phenyl linker showed the opposite performance, and disubstituted **CC2TA** revealed better performances (11%) than the monosubstituted **CzT** molecule (6%). Symmetrical molecules of **DPA-TRZ** and **DACT-II** with a phenyl linker unit exhibited better device properties. Carbazole with diphenyl amine at the third and sixth positions (**DACT-II)** enhanced the device quantum efficiency up to 29.6%, while diphenyl amine, at the third and sixth positions of diphenylamine (**DPA-TRZ)**, showed a low efficiency of 13.8%. So, carbazole with a diphenylamine donor at the third and sixth positions resulted in a more interesting effect with the triazine acceptor than a similar molecular design with diphenylamine donor derivatives. A **IDCzTrzDBF** molecule was constructed with a furan linker between an indolocarbazole donor and a triazine acceptor, but this linker moiety and substituted position did not have a successful effect on the external quantum efficiency. A furan linker moiety attached to a symmetrical donor of carbazole with third- and sixth-position-substituted phenyl carbazole (**TCzTrzDBF**) showed better performance. When the number of carbazole donor moieties was increased and attached to the phenyl group at meta and para positions (**TmCzTrz**), the result was an EQE over 25%. Overall, a carbazole donor containing a symmetrical structure, along with free rotating substitutions at the third and sixth positions, and a number of carbazole donors attached through meta and para substitution further helped to achieve high EQEs for green TADF emitters compared with carbazole-based rigid donor derivatives of indolocarbazole. 

The indeno–acridine strong-donor-based 5-(4-(4,6-diphenyl-1,3,5-triazin-2-yl)phenyl)-7,7,13,13-tetramethyl-7,13-dihydro-5H-indeno[1,2-b]acridine (**TrzIAc**) molecule (Figure 3) was reported to have a PLQY of 97%. Delayed fluorescence was observed at 1.6 μs, with a singlet–triplet energy difference of 0.06 eV. The rigid donor molecule enhanced the thermal stabilities of **TrzIAc**. OLED device performances were noticed when 20 wt % was doped with mixed hosts of mCP and TPBI. An EQE of 20.9% was recorded, which was higher than that of acridine-donor-based **TrzAc** (17.7%). The indeno–acridine donor moiety not only enhanced the thermal stability but also improved device efficiencies with green color emission (511 nm) [61]. 

Kang et al. reported two TADF emitters with rigid donors of benzofuran–acridine (13-(4-(4,6-diphenyl-1,3,5-triazin-2-yl)phenyl)-5,5-dimethyl-5,13-dihydrobenzofuro[3,2-c]acridine, **BFAcTRZ**) and benzothiophene–acridine (13-(4-(4,6-diphenyl-1,3,5-triazin-2-yl)phenyl)-5,5-dimethyl-5,13-dihydrobenzo[4,5]thieno[3,2-c]acridine, **BTAcTRZ**) with a triazine acceptor (Figure 3). The PLQY of **BTAcTRZ** was as high as 100%, and the energy difference between singlet and triplet states was as small as 0.02 eV, with a delayed fluorescence of 9.3 μs. The constant of reverse intersystem crossing was higher in benzothiophene-based **BTAcTRZ**. The EQEs of **BFAcTRZ** (20.4%) and **BTAcTRZ** (21.8%) were higher than 20%, and **BTAcTRZ** exhibited better efficiencies than that of the indeno–acridine-based **TrzIAc** molecule. The **BTAcTRZ** molecule showed a red-shifted electroluminescent emission of 526 nm due to its donor moiety [61,92].

Rigid donor moieties of indeno–acridine-, dibenzofuro–acridine-, and benzothieno–acridine-based **TrzIAc**, **BFAcTrz**, and **BTAcTrz** revealed better EQEs, which were higher than those of indolocarbazole-based green TADF emitters.

The 10-(4-(4,6-diphenyl-1,3,5-triazin-2-yl)phenyl)-10H-phenoxazine (**PXZ-TRZ**) material with a phenoxazine donor (Figure 4) showed a dihedral angle between donor and acceptor of 74.8°, which made an effective separation of HOMO and LUMO. There was a small energy gap between the singlet and triplet states of 0.07 eV, which was obtained through a phenyl linker between the donor and the acceptor. A PLQY of 65.7% resulted in an EQE of 12.5%. There was a short delayed fluorescence at 0.68 μs, and the maxima of the electroluminescent spectra was at 529 nm. Later, di- and trisubstituted phenoxazine donors with a triazine acceptor moiety were reported. 2,4,6-Tris(4-(10H-phenoxazin-10-yl)phenyl)-1,3,5-triazine (**Tri-PXZ-TRZ**) revealed an EQE of 13.3%, which was higher than that of the single-substituted donor molecule. Moreover, the trisubstituted material showed red-shifted emission, and the PLQY was 58% [93,94].

Tanaka et al. developed the phenothiazine-donor-and-triazine-acceptor-based 10-(4-(4,6-diphenyl-1,3,5-triazin-2-yl)phenyl)-10H-phenothiazine (**PTZ-TRZ**) TADF emitter (Figure 5), which had a phenyl linker between the donor and the acceptor. It was noticed that **PTZ-TRZ** exhibited dual ICT fluorescence in solid and solution states with a small singlet–triplet energy difference. **PTZ-TRZ** revealed quasi-equatorial conformation and had a lower dihedral angle between donor and acceptor compared with phenoxazine-based **PXZ-TRZ**. Two PL emissions were observed at 409 and 562 nm, which were assigned to the quasi-axial and quasi-equatorial conformers, respectively. This OLED device, at a low concentration of 2 wt % **PTZ-TRZ**, showed an EQE of 10.8% and a PLQY of 65.8% [60,93].

Shizu et al. reported another molecule (N1-(4-(4,6-diphenyl-1,3,5-triazin-2-yl)phenyl)-N1-(4-(diphenylamino)phenyl)-N4,N4-diphenylbenzene-1,4-diamine, **DPA-TRZ**) (Figure 5) with a 100% PLQY. When **DPA-TRZ** was doped with host material, nonradiative decay was suppressed, which helped the effective reverse intersystem crossing mechanism. A long delayed component of 160 μs was observed, which confirmed the presence of TADF characteristics. An EQE of 13.8% was recorded at a current density of 0.01 mA/Cm^−2^. The device efficiencies were notably better than those of phenothiazine-based **PTZ-TRZ** and phenoxazine-based **PXZ-TRZ** devices. The electroluminescent (EL) emission was notices at 548 nm, which showed little red shifting due to its long conjugation donor molecule [60,93,95]. 

Tsai et al. introduced 10-(4-(4,6-diphenyl-1,3,5-triazin-2-yl)phenyl)-9,9-dimethyl-9,10-dihydroacridine (**DMAC-TRZ**) (Figure 6) with a dimethyl acridine donor, which showed high PLQYs of 83% and 90% for neat and doped films, respectively. The geometrical optimization showed that **DMAC-TRZ** had a large dihedral angle of 88° between the acridine donor and the triazine acceptor. **DMAC-TRZ** exhibited more stable thermal properties than those of phenoxazine-based **PXZ-TRZ.** Delayed fluorescence was observed at 3.6 μs in the neat film state, while it was 1.9 μs in the doped film state. The high PLQY in neat and doped film states suggests that two different devices with doped and undoped TADF emitters could be fabricated. The doped device with mCPCN host material showed current, power, and external quantum efficiencies of 66.8 cd/A, 65.6 lm/W, and 26.5%, respectively. The undoped device had current and external quantum efficiencies of 61.1 cd/A and 20%. Such high efficiencies for an undoped device were explained by the effective mechanism of RISC [82,93].

Further development of acridine-donor-based 2,4,6-tris(4-(9,9-dimethylacridin-10(9H)-yl)phenyl)-1,3,5-triazine (**3ACR-TRZ**) TADF emitters (Figure 6) for solution-processable OLEDs was reported by Wada et al. **3ACR-TRZ** showed a high PLQY of 98%, which was higher than that of **DMAC-TRZ**. The increased number of acridine donor molecules helped to reduce the energy gap between the singlet and triplet states to 0.015 eV, and a slightly longer delayed fluorescence was recorded at 6.7 μs. The OLED device was fabricated with a 16 wt % emitter doped with CBP host material. The EQE was 18.6%, which was higher than that of the phenoxazine-based three site molecule **Tri-PXZ-TRZ**. The dimethyl acridine donor provided good properties as well as easy solution processability [82,94,96].

Recently, our group reported two TADF emitters based on an acridine–triazine molecular backbone. Two different acridine donors, such as dimethyl acridine (10,10'-(5-(4,6-diphenyl-1,3,5-triazin-2-yl)-1,3-phenylene)bis(9,9-dimethyl-9,10-dihydroacridine), **TRZ-DDMAc**) and diphenyl acridine (10,10’-(5-(4,6-diphenyl-1,3,5-triazin-2-yl)-1,3-phenylene)bis(9,9-diphenyl-9,10-dihydroacridine), **TRZ-DDPAc**) were constructed (Figure 6) with a D-A-D structure and a phenyl linker between the donor and the acceptor. Interestingly, the diphenyl-acridine-donor-based molecule revealed a PLQY (79.7%) higher than that of the dimethyl-acridine-based molecule. The calculation method showed that the nonradiative decay rate of the dimethyl acridine donor molecule was two times that of the diphenyl donor molecule. A device using **TRZ-DDPAc** doped with the polar host material DBFPO showed excellent efficiencies of 62.8 cd/A, 56.3 lm/W, and 27.3% for current, power, and external quantum efficiencies, respectively [83].

Regarding the device performances of triazine-based green TADF emitters, **DACT-II**-, **TRZ-DDPAc**-, **DMAC-TRZ**-, and **TmCzTrz**-based devices exhibited good EQEs of 29.6%, 27.3%, 26.5%, and 25.5%, respectively. The **DACT-II** molecular design had a carbazole donor with symmetrically attached diphenylamine units, while the **TmCzTrz** molecule was constructed with three carbazole units at the meta and para positions of the phenyl linker. **TRZ-DDPAc** had two diphenyl acridine moieties at the meta positions of the phenyl linker group. The molecular design containing only donors of phenoxazine and phenothiazine did not show any impact on efficiency enhancement. Acridine derivatives had prominent effects on quantum efficiencies. The number of donor moieties with symmetrical attachments with the linker phenyl group enhanced the device properties.

Many donor derivatives were incorporated with triazine acceptors to design various green TADF emitters. The strong donor moieties of acridine, phenothiazine, diphenylamine, and phenoxazine and the weak donor moieties of carbazole, benzofurocarbazole, and benzothioenocarbazole were employed, showing suitable attachment to acceptor and linker units. We noticed that strong-donor-based **PXZ-TRZ**, **PTZ-TRZ**, **Bis-PXZ-TRZ**, **Tris-PXZ-TRZ**, and **DPA-TRZ** showed red-shifted emissions of 529, 532, 552, 553, and 548 nm, respectively. The carbazole-donor-based **CC2TA**, **PIC-TRZ**, **PIC-TRZ2**, **DCzmCzTrz**, and **TmCzTrz** showed blue-shifted emissions of 490, 500, 505, 496, and 500 nm, respectively. So, future works should consider elucidating which type of donor moiety is suitable for designing green TADF emitters with color purity emission. 

Moreover, the selection of host materials, adjacent layers, and doping concentrations is important to ensure the effectiveness of the device. Among the above-reported triazine-based green TADF emitters, 20 wt % doped emitters of **TrzIAc** and **DCzmCzTrz** showed EQEs of 20.9% and 21.3%, respectively, and an emission layer thickness of 25 nm. The 30 wt % doped **BFAcTrz**, **TmCzTrz**, and **TRZ-DDPAc** exhibited quantum efficiencies of 20.4%, 25.5%, and 27.3%, respectively, and had the same emission layer thickness of 25 nm. A **DACT-II**-based device showed better device properties at a low doping concentration of 6%, but the thickness of the emission layer was reported to be 40 nm, and we believe that the greater thickness of the host material (CBP) supported an effective energy flow to achieve an EQE of 29.6%. **DMAC-TRZ** showed good device characteristics (EQE of 26.5%) and employed an 8% doping (20 nm) concentration and hole-blocking layer (DPSS). The acridine-based **3ACR-TRZ** was 16 wt % doped with CBP as the host material, and the emission layer thickness was as high as 55 nm, but the device could not reach an EQE over 19%. So, for device optimization, using various doping concentrations and host materials is crucial to obtain an effective device. Host materials play a major role in device performance as they are responsible for supplying energy to the emission layer. At the same time, host materials control the charge recombination of collected electrons and holes from the cathode and anode, respectively. The choice of host material depends on the triplet energy of the dopant material, and high triplet energy host materials dope with dopant to establish an effective device. 

## 3. Conclusions

Triazine-acceptor-based green TADF emitters with suitable donor derivatives and host materials have shown great performance in terms of device efficiency. The EQEs were over 29%, which were higher than those of any red TADF emitters. Still, many improvements are needed in the molecular design to achieve a high efficiency. Host materials play a major role in device efficiency by supporting effective energy transfer to the dopant. Moreover, a proper doping concentration also enhances device performance. Triazine has exhibited good withdrawing characteristics and a suitable donor moiety connecting the appropriate position, which should result in a highly efficient and stable molecular design for green TADF emitters.

## Figures and Tables

**Table 1:** Photophysical properties of triazine-based green thermally activated delayed fluorescence (TADF) emitters.

TADF Emitter	HOMO (eV)	LUMO (eV)	PL (nm)	∆E_ST_ (eV)	Φ_PL_ (%)	τ_d_ (μs)	Reference
**PIC-TRZ**	-	-	500	0.11	39	230	[86]
**PXZ-TRZ**	5.5	3.1	540	0.07	65.7	0.68	[89]
**CC2TA**	5.9	2.6	513^a^	0.05	62	22	[87]
**PIC-TRZ2**	-	-	-	0.02	45	2.7	[86]
**Bis-PXZ-TRZ**	5.7	3.4	560 ^a^	0.054 ^b^	64	1.33 ^a^	[90]
**Tri-PXZ-TRZ**	5.7	3.4	568 ^a^	0.065 ^b^	58	1.10 ^a^	[90]
**CzT**	-	-	502	0.07	39.7	42.6 ^a^	[88]
**PTZ-TRZ**	5.5	3.0	420,520	0.18 ^b^	65.8	0.52 ^a^	[89]
**DMAC-TRZ**	5.3	2.78	510	0.05	83	3.6	[82]
**DACT-II**	5.5	3.2	520	0.009	100	-	[84]
**DPA-TRZ**	-	-	540	0.11	100	160	[91]
**TrzIAc**	5.75	3.34	519	0.06	97 ^a^	1.6	[61]
**3ACR-TRZ**	-	-	504	0.015	98 ^a^	6.7	[93]
**BCzTrzDBF**	5.85	3.34	-	0.06	82.4	5.4	[95]
**TCzTrzDBF**	5.87	3.43	-	0.01	86.3	4.4	[95]
**IDCzTrzDBF**	5.88	3.34	-	0.05	85.4	2.8	[95]
**BFAcTrz**	5.84	3.23	-	0.11	92.3	14.2	[94]
**BTAcTrz**	5.8	3.24	-	0.02	100	9.3	[94]
**TmCzTrz**	5.19 ^b^	2.11 ^b^	-	0.07	100	13.3	[92]
**DCzmCzTrz**	5.26 ^b^	2.15 ^b^	-	0.20	98	9.7	[92]
**TRZ-DDMAc**	5.70	2.89	529	0.03	52.7	10.32	[83]
**TRZ-DDPAc**	5.72	2.87	511	0.05	79.7	10.37	[83]
**2Cz3Trz**	5.78	3.32	-	0.06	74.2	4.80	[96]
**3Cz2Trz**	5.77	3.20	-	0.05	69.7	2.84	[96]

^a^ Measured in solution state. ^b^ Calculation value.

**Table 2:** Organic light-emitting diodes (OLED) device evaluation performances of triazine-based green TADF emitters.

TADF Emitter	Device Structure	EML (nm)	EL_max_ (nm)	CIE Color	CE (cd/A)	PE (lm/W)	EQE (%)	Reference
**PIC-TRZ**	ITO/NPD/α-mCP/6 wt % **PIC-TRZ**: mCP/BP4mPy/LiF/Al	15	500	-	-	-	5.3	[86]
**PXZ-TRZ**	ITO/α-NPD/6 wt % **PXZ-TRZ**: CBP/TPBi/LiF/Al	15	529	-	-	-	12.5	[89]
**CC2TA**	ITO/α-NPD/6 wt % CC2TA: mCP/6 wt % **CC2TA**: DPEPO/DPEPO/TPBi/LiF/Al	30	490	-	-	-	11.0	[87]
**PIC-TRZ2**	ITO/TAPC/6 wt % **PIC-TRZ2**: PYD2/DPEPO/TmPyPBi/LiF/Al	20	505	-	-	-	14.0	[86]
**Bis-PXZ-TRZ**	ITO/α-NPD/6 wt % **Bis-PXZ-TRZ**: mCBP/TPBi/LiF/Al	15	552	-	-	-	9.1	[90]
**Tri-PXZ-TRZ**	ITO/α-NPD/6 wt % **Tri-PXZ-TRZ**: mCBP/TPBi/LiF/Al	15	553	-	-	-	13.3	[90]
**CzT**	ITO/α-NPD/TCTA/CzSi/3 wt % **CzT**: DPEPO/DPEPO/TPBi/LiF/Al	20	520	0.23, 0.40	-	9.7	6.0	[88]
**PTZ-TRZ**	ITO/α-NPD/2 wt % **PTZ-TRZ**: mCBP/TPBi/LiF/Al	15	532	-	-	-	10.8	[89]
**DMAC-TRZ**	ITO/PEDOT: PSS/TAPC/mCP/8 wt % **DMAC-TRZ**: mCPCN/DPSS/3TPYMB/LiF/Al (nondoped device): ITO/PEDOT: PSS/TAPC/mCp/**DMAC-TRZ**/3TPYMB/LiF/Al	20	-	-	66.8 61.1	65.6 45.7	26.5 20.0	[82]
**DACT-II**	ITO/TAPC/9 wt % **DACT-II**: CBP/BAlq/Liq/Al	40	-	-	-	-	29.6	[84]
**DPA-TRZ**	ITO/α-NPD/6 wt % **DPA-TRZ**: mCBP/TPBi/LiF/Al	15	548	-	-	-	13.8	[91]
**TrzIAc**	20 wt % **TrzIAc**: mCP and TPBI	25	511	0.33, 0.57	-	-	20.9	[61]
**3ACR-TRZ**	ITO/PEDOT: PSS/16 wt % **3ACR-TRZ**: CBP/BmPyPhB/Liq/Al	55	520	-	-	-	18.6	[93]
**BCzTrzDBF**	ITO/DNTPD/BPBPA/PCzAc/5 wt % **BCzTrzDBF**: mCBPTrz/DBFTrz/ZADN/LiF/Al	30	503	0.24, 0.52	59.6	35.1	20.1	[95]
**TCzTrzDBF**	ITO/DNTPD/BPBPA/PCzAc/5 wt % **TCzTrzDBF**: mCBPTrz/DBFTrz/ZADN/LiF/Al	30	511	0.27, 0.57	74.8	44.7	23.5	[95]
**IDCzTrzDBF**	ITO/DNTPD/BPBPA/PCzAc/5 wt % **IDCzTrzDBF**: mCBPTrz/DBFTrz/ZADN/LiF/Al	30	500	0.22, 0.48	33.6	19.3	12.2	[95]
**BFAcTrz**	ITO/PEDOT: PSS/TAPC/mCP/30 wt % **BFAcTrz**: DPEPO/TSPOI/TPBi/LiF/Al	25	506	0.25, 0.51	58.7	52.7	20.4	[94]
**BTAcTrz**	ITO/PEDOT: PSS/TAPC/mCP/50 wt % **BTAcTrz**: DPEPO/TSPO1/TPBi/LiF/Al	25	526	0.35, 0.57	68.9	58.5	21.8	[94]
**TmCzTrz**	ITO/PEDOT: PSS/TAPC/mCP/30 wt % **TmCzTrz**: DPEPO/TSPO1/TPBI/LiF/Al	25	500	0.25, 0.50	18.6	52.1	25.5	[92]
**DCzmCzTrz**	ITO/PEDOT: PSS/TAPC/mCP/20 wt % **DCzmCzTrz**: DPEPO/TSPO1/TPBI/LiF/Al	25	496	0.23, 0.46	16.8	42.4	21.3	[92]
**TRZ-DDPAc**	ITO/HATCN/TAPC/DCDPA/30 wt % **TRZ-DDPAc**: DBFPO/TPBi/LiF/Al	25	509	0.25, 0.52	62.8	56.3	27.3	[83]
**TRZ-DDMAc**	ITO/HATCN/TAPC/DCDPA/20 wt % **TRZ-DDMAc**: PPBI/TPBi/LiF/Al	25	511	0.26, 0.54	43.2	33.7	17.6	[83]
**2Cz3Trz**	ITO/DNTPD/BPBPA/PCZAC/10 wt % **2Cz3Trz**: CzTrz/CzTrz/ZADN/LiF/Al	30	521	0.30, 0.56	55.8	28.3	17.9	[96]
**3Cz2Trz**	ITO/DNTPD/BPBPA/PCZAC/10 wt % **3Cz2Trz**: CzTrz/CzTrz/ZADN/LiF/Al	30	512	0.26, 0.50	42.9	21.8	15.0	[96]
